# Coupled Information–Epidemic Spreading Dynamics with Selective Mass Media

**DOI:** 10.3390/e25060927

**Published:** 2023-06-12

**Authors:** Jiajun Xian, Zhihong Zhang, Zongyi Li, Dan Yang

**Affiliations:** Department of Computer Science, School of Engineering, Shantou University, Shantou 515063, China

**Keywords:** multiplex network, epidemic spreading, information dissemination, mass media

## Abstract

As a pandemic emerges, information on epidemic prevention disseminates among the populace, and the propagation of that information interacts with the proliferation of the disease. Mass media serve a pivotal function in facilitating the dissemination of epidemic-related information. Investigating coupled information–epidemic dynamics, while accounting for the promotional effect of mass media in information dissemination, is of significant practical relevance. Nonetheless, in the extant research, scholars predominantly employ an assumption that mass media broadcast to all individuals equally within the network: this assumption overlooks the practical constraint imposed by the substantial social resources required to accomplish such comprehensive promotion. In response, this study introduces a coupled information–epidemic spreading model with mass media that can selectively target and disseminate information to a specific proportion of high-degree nodes. We employed a microscopic Markov chain methodology to scrutinize our model, and we examined the influence of the various model parameters on the dynamic process. The findings of this study reveal that mass media broadcasts directed towards high-degree nodes within the information spreading layer can substantially reduce the infection density of the epidemic, and raise the spreading threshold of the epidemic. Additionally, as the mass media broadcast proportion increases, the suppression effect on the disease becomes stronger. Moreover, with a constant broadcast proportion, the suppression effect of mass media promotion on epidemic spreading within the model is more pronounced in a multiplex network with a negative interlayer degree correlation, compared to scenarios with positive or absent interlayer degree correlation.

## 1. Introduction

The dissemination of epidemic-related information can effectively stimulate individuals to cultivate a heightened sense of precaution, subsequently prompting them to adopt protective measures to mitigate the spread of the epidemic. Conversely, the spreading of an epidemic can foster the diffusion of pertinent information within populations, thereby augmenting collective protective awareness. For instance, following the emergence of the COVID-19 pandemic, corollary information began disseminating throughout the internet. Upon acquiring the COVID-19-related information, individuals engaged in a myriad of self-protective behaviors, including the utilization of masks, frequent hand-washing, and adherence to social distancing measures [1,2,3,4,5,6,7,8]. Such self-protective actions served to disrupt the transmission pathways of the virus, ultimately curbing the outbreak of the epidemic [9,10,11,12,13,14,15,16]. Investigation of the coupled propagation of an epidemic and its associated information has consistently piqued the interest of scholars, and is of considerable academic significance [17,18,19,20,21,22,23,24,25,26].

A multitude of researchers have employed a dual-layer network structure, to establish coupled information–epidemic propagation models, aiming to expound upon the reciprocal relationship between the spread of epidemic-related information and the proliferation of the epidemic itself [27,28,29,30,31,32,33,34,35,36]. Within this framework, the lower-layer network represents the epidemic spreading stratum, with nodes symbolizing individuals, and edges signifying physical contact relationships in reality. Simultaneously, the upper-layer network constitutes the information dissemination stratum, with nodes corresponding to the same individuals as the lower layer, and edges illustrating information exchange relationships amongst them [37,38,39,40,41,42,43,44,45,46]. Granell et al. introduced a coupled information–epidemic propagation model based on dual-layer networks, which delineated the interrelated transmission phenomena of an epidemic and its associated information within the real world [47]. Granell et al. discovered the existence of a metacritical point, at which the spreading threshold of the epidemics started depending on the information dissemination [47]. Building upon this work, Guo et al. proposed a disease epidemic spreading model that accounted for local awareness, investigating epidemic spreading accompanied by awareness cascades. Guo et al. ascertained that the epidemic spreading threshold experienced an abrupt alteration when the local awareness rate approached a specific value. These discoveries have contributed to a more profound comprehension of decision-making processes predicated on the behaviors of others, as well as an understanding of why certain epidemics fail to manifest in reality [48]. Moreover, acknowledging that individuals may access epidemic information from a diverse array of channels in real-life scenarios, numerous scholars have undertaken comprehensive research on the repercussions of multi-source information dissemination on epidemics within the dual-layer network. Xiao et al., for example, put forth a coupled information–disease propagation model, incorporating time-varying self-awareness and behavioral responses, employed to assess the impact of dynamic multi-source information and behavioral responses on the coupling of epidemics and information within time-varying multi-layer networks. They concluded that the efficacy of dynamic multi-source information in impeding epidemic spreading remains circumscribed, and that time-varying self-awareness and behavioral responses will exert a considerable influence on suppressing epidemic spreading only when a majority of individuals can access multi-source information, and implement corresponding behavioral reactions [49]. In addition, Li et al. also proposed that, in addition to online information dissemination, offline interaction and information spreading in person can be important for epidemic intervening, and can be better analyzed in spatio-temporal interaction networks [50]. Furthermore, Li et al. found that the nature of connection in a multiplex network [51] and in a community structure [52] can strongly affect the spreading dynamics.

In recent years, with the rapid development of internet technology, mass media (for instance, the written and digital press, radio, and television) has greatly improved the efficiency of information dissemination, playing an increasingly important role in the coupled propagation of epidemics and their related information. Consequently, scholars have incorporated the impact of mass media into their research on coupled information–disease propagation. Granell et al. found that mass media could indirectly influence the outbreak size of epidemics, by interfering with the dissemination of epidemic-related information, thus increasing the spreading threshold of the epidemics [53]. Ma et al. analyzed the effects of self-awareness and mass media on the spreading of epidemics, discovering that a reduction in the proportion of asymptomatic carriers would suppress epidemic spreading, and increase the epidemic spreading threshold [54]. Wang et al. studied the coupled propagation of multi-types information and epidemics under the influence of mass media, finding that accelerating the spread of positive information by strengthening media publicity could effectively suppress epidemic spreading, and that when positive information was not disseminated, accelerating the spread of negative information could also alleviate the occurrence of epidemics [55].

In summary, research on the coupled propagation of epidemics and their associated information that considers the impact of mass media has important practical significance, and has achieved notable research results. However, in previous studies, scholars have generally assumed that mass media engage in undifferentiated promotion efforts, targeting all individuals within a network, neglecting the fact that such comprehensive promotion requires substantial social resources, and is difficult to achieve; therefore, this study proposes a coupled information–disease propagation model, incorporating a selective promotion mechanism for mass media. Within this model, mass media can selectively target a specific proportion of high-degree nodes, for dissemination. We also developed a microscopic Markov chain method for model analysis, and we discuss the influence of different model parameters on the dynamic evolution process.

The organization of this paper is as follows. In Section 2, we describe in detail our proposed coupled information–disease propagation model with its selective promotion mechanism for mass media. In Section 3, we introduce how to develop a microscopic Markov chain method for the analysis of our model, deriving the final transmission scope and threshold of the disease. For Section 4, we used Monte Carlo simulations (MC) to verify the accuracy of the theoretical predictions, and we analyzed the impact of the model parameters on the coupled dynamics. In Section 5, we summarize the paper, and provide an outlook for future research.

## 2. Model Description

**Figure 1:**
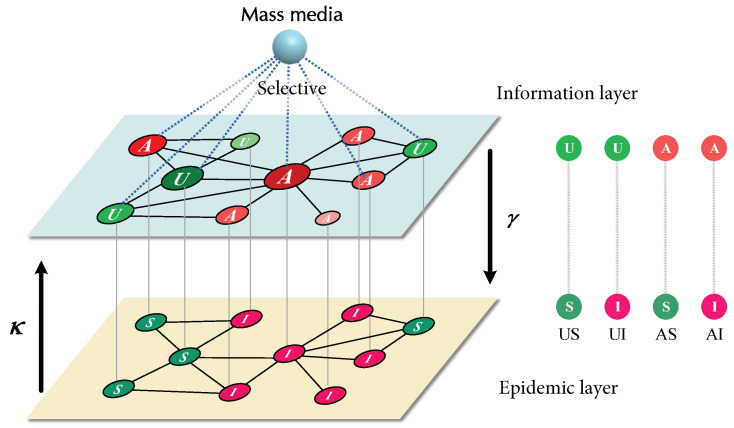
Coupled information–epidemic spreading dynamics with selective mass media. The upper (information) layer represents the virtual-contact network, which supports the dissemination of information. Nodes have two possible states in the upper layer: unaware (U) and aware (A). The lower (epidemic) layer represents the physical-contact layer, which supports the epidemic spreading. Nodes in the lower layer correspond one-to-one with nodes in the upper layer, but their possible states are: susceptible (S) and infected (I). In addition, a global node representing selective mass media is also utilized, which is connected to all nodes, and can selectively broadcast information to nodes.

This section will introduce the proposed coupled information–epidemic spreading dynamics with selective mass media. As shown in Figure 1, the coupled information–disease propagation model is built on a two-layer multiplex network framework. Within this model, the epidemic occurs in a physical-contact layer, while epidemic-related information spreads through a virtual-contact layer. Although the nodes in the two network layers have a one-to-one correspondence, their connectivity is unique, resulting in distinct topologies for each layer. Based on this two-layer multiplex network framework, we incorporated a Susceptible-Infected-Susceptible (SIS) process into the physical-contacts layer. Here, the probability of a susceptible node becoming infected after contact with an infected node was denoted by β, while the likelihood of an infected node recovering spontaneously was represented by μ. For the virtual-contact layer, we applied a similar process, called Unaware-Aware-Unaware (UAU), to describe the information diffusion. Here, each node could be in two states: Aware (A) and Unaware (U). Unaware nodes could receive information from aware neighbors, with a probability of λ. Meanwhile, aware nodes could forget the information, and become unaware, with a probability of δ.

The evolutionary processes of these two processes interact with each other. Specifically, when a node is infected in the SIS process, it will become aware of the infection in the UAU information layer, with a probability of κ. In addition, the SIS process’ infectivity rate depends on the node’s awareness in the UAU information layer. We denoted $\beta^{U}$ as the infection probability for an unaware node, while $\beta^{A}$ = γ$\beta^{U}$ represented the probability for an aware node. The γ parameter varied from 0 (complete immunity) to 1 (information awareness that had no impact on the epidemic). It is important to note that when γ = 1 and κ=0, both interactions are deactivated, making the system equivalent to running separate single-layer networks for each process.

In addition to the two interactive processes within the model, a global node representing mass media with a selective publicity mechanism is also utilized. This node is connected to all nodes in the UAU information layer, and can selectively broadcast information to nodes ranked within mass media broadcast proportion η. The probability of an unaware node becoming aware, after being exposed to mass media propaganda, is denoted as *m*. Consequently, in the UAU information layer, at each time step, any node can acquire information through the UAU process, while nodes with degree rankings within η can also access information via mass media.

## 3. Theoretical Analysis

### 3.1. Microscopic Markov Chain Approach

**Figure 2:**
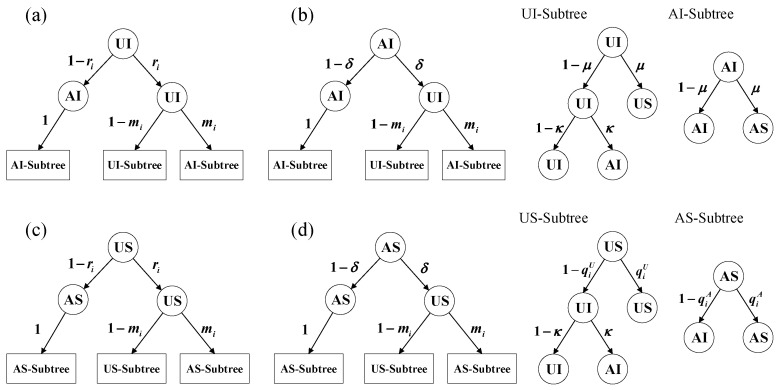
Transition probability trees for four possible node states in the coupled information–epidemic spreading model with selective mass media. Subfigures (**a**–**d**) represent the transition probability trees of different node states UI, AI, US and AS, respectively. The roots of the trees represent the possible states of each node at time *t*, and the leaves represent its possible states at time t+1. Each time step can be divided into four continuous phases: information dissemination(UAU process); mass media broadcasting; self-awareness of being infected; and epidemic spreading (SIS process).

In this section, we employ the microscopic Markov chain approach (MMCA), to analytically derive our model. Based on the assumptions of the model, there are four possible states for each node in the multiplex network: unaware and susceptible (US); aware and susceptible (AS); unaware and infected (UI); aware and infected (AI). Figure 2 shows the state transition probability trees of the nodes, which account for all potential state changes and their associated probabilities at each time step.

During each time step, the evolution of node states can be categorized into four stages: (I) the awareness dissemination stage, i.e., the UAU process; (II) the mass media publicity stage; (III) the epidemic transmission stage, i.e., the SIS process; and (IV) the self-awakening stage for infected nodes. For simplicity, we denote $f_{i}^{X\to Y},X,Y\in \left(U,A\right)$ as the probability of node change from state X to Y after stage (I) and (II), and we can obtain

(1) $$\begin{array}{c} \begin{array}{c} f_{i}^{U\to U}=r_{i}\left(t\right)\left(1-m_{i}\right), \end{array} \end{array}$$

(2) $$\begin{array}{c} \begin{array}{c} f_{i}^{A\to U}=\delta \left(1-m_{i}\right), \end{array} \end{array}$$

(3) $$\begin{array}{c} \begin{array}{c} f_{i}^{A\to A}=1-\delta +\delta m_{i}, \end{array} \end{array}$$

and

(4) $$\begin{array}{c} \begin{array}{c} f_{i}^{U\to A}=1-r_{i}\left(t\right)+r_{i}\left(t\right)m_{i}. \end{array} \end{array}$$

In this context,

(5) $$\begin{array}{c} r_{i}\left(t\right)=\prod_{j}\left[1-a_{ji}p_{j}^{A}\left(t\right)\lambda \right] \end{array}$$

represents the probability that node *i* does not transition from the U state to the A state during the UAU process, where $p_{i}^{A}\left(t\right)$ denotes the probability that node *j* is in the A state at time *t*, and $a_{ji}$ represents the elements of the adjacency matrix of the information spreading layer. In addition, $m_{i}\left(t\right)$ represents the probability that node *i* shifts from the U state to the A state during stage (II), as a result of mass media influence—specifically:

(6) $$\begin{array}{c} m_{i}=\left\{\begin{array}{c} m,\;\eta >\frac{R_{i}}{N}\\ 0,\;\eta \leq \frac{R_{i}}{N} \end{array}\right., \end{array}$$

where *N* is the number of nodes, and $R_{i}$ is the degree ranking of node *i* among all the nodes. Equation (6) indicates that only nodes with degree rankings within η can access information via mass media.

From the transition trees in Figure 2, we can recover the MMCA equations representing the state probability evolution of each node, as follows:

(7) $$\begin{array}{c} \begin{array}{cc} p_{i}^{\mathrm{US}}\left(t+1\right) & =p_{i}^{\mathrm{UI}}\left(t\right)f_{i}^{U\to U}\mu +p_{i}^{\mathrm{AI}}\left(t\right)f_{i}^{A\to U}\mu \\ & +p_{i}^{\mathrm{US}}\left(t\right)f_{i}^{U\to U}q_{i}^{U}\left(t\right)+p_{i}^{\mathrm{AS}}\left(t\right)f_{i}^{A\to U}q_{i}^{U}\left(t\right), \end{array} \end{array}$$

(8) $$\begin{array}{c} \begin{array}{cc} p_{i}^{\mathrm{UI}}\left(t+1\right) & =p_{i}^{\mathrm{UI}}\left(t\right)f_{i}^{U\to U}\left(1-\mu \right)\left(1-\kappa \right)\\ & +p_{i}^{\mathrm{AI}}\left(t\right)f_{i}^{A\to U}\left(1-\mu \right)\left(1-\kappa \right)\\ & +p_{i}^{\mathrm{US}}\left(t\right)f_{i}^{U\to U}\left[1-q_{i}^{U}\left(t\right)\right]\left(1-\kappa \right)\\ & +p_{i}^{\mathrm{AS}}\left(t\right)f_{i}^{A\to U}\left[1-q_{i}^{U}\left(t\right)\right]\left(1-\kappa \right), \end{array} \end{array}$$

(9) $$\begin{array}{c} \begin{array}{cc} p_{i}^{\mathrm{AS}}\left(t+1\right) & =p_{i}^{\mathrm{UI}}\left(t\right)f_{i}^{U\to A}\mu +p_{i}^{\mathrm{AI}}\left(t\right)f_{i}^{A\to A}\mu \\ & +p_{i}^{\mathrm{US}}\left(t\right)f_{i}^{U\to A}q_{i}^{A}\left(t\right)+p_{i}^{\mathrm{AS}}\left(t\right)f_{i}^{A\to A}q_{i}^{A}\left(t\right), \end{array} \end{array}$$

and

(10) $$\begin{array}{c} \begin{array}{cc} p_{i}^{\mathrm{AI}}\left(t+1\right) & =p_{i}^{\mathrm{UI}}\left(t\right)f_{i}^{U\to A}\left(1-\mu \right)+f_{i}^{U\to U}\left(1-\mu \right)\kappa \\ & +p_{i}^{\mathrm{AI}}\left(t\right)f_{i}^{A\to A}\left(1-\mu \right)+f_{i}^{A\to U}\left(1-\mu \right)\kappa \\ & +p_{i}^{\mathrm{US}}\left(t\right)f_{i}^{U\to A}\left[1-q_{i}^{A}\left(t\right)\right]+f_{i}^{U\to U}\left[1-q_{i}^{U}\left(t\right)\right]\kappa \\ & +p_{i}^{\mathrm{AS}}\left(t\right)f_{i}^{A\to A}\left[1-q_{i}^{A}\left(t\right)\right]+f_{i}^{A\to U}\left[1-q_{i}^{U}\left(t\right)\right]\kappa , \end{array} \end{array}$$

where

(11) $$\begin{array}{c} q_{i}^{A}\left(t\right)=\prod_{j}\left[1-b_{ji}p_{j}^{I}\left(t\right)\beta^{A}\right] \end{array}$$

and

(12) $$\begin{array}{c} q_{i}^{U}\left(t\right)=\prod_{j}\left[1-b_{ji}p_{j}^{I}\left(t\right)\beta^{U}\right] \end{array}$$

represents the probabilities of node *i* not being infected by any neighbor in the SIS process, when *i* is in states A and U, respectively.

By iterating Equations (7) to (10), the stationary state of the full system can be obtained from any initial condition.

### 3.2. Threshold Analysis

Based on the MMCA equations of the system, we can proceed to analytically derive the epidemic threshold $\beta_{c}^{U}$. Epidemics can break out when $\beta^{U}>\beta_{c}^{U}$, and will die out otherwise.

The fraction of nodes in state I can be calculated as

(13) $$\begin{array}{c} \rho^{I}=\frac{1}{N}\sum_{i=1}^{N}p_{i}^{I}=\frac{1}{N}\sum_{i=1}^{N}\left(p_{i}^{\mathrm{UI}}+p_{i}^{\mathrm{AI}}\right). \end{array}$$

When the system reaches the stationary state, i.e., t→∞, we have $p_{i}^{\mathrm{XY}}\left(t+1\right)=p_{i}^{\mathrm{XY}}\left(t+1\right)=p_{i}^{\mathrm{XY}},X,Y\in \left(U,A\right)$ for all node *i*: thus, it can be obtained that

(14) $$\begin{array}{c} \begin{array}{cc} p_{i}^{I} & =p_{i}^{I}\left(1-\mu \right)\\ & +p_{i}^{\mathrm{US}}\left\{r_{i}\left(1-m_{i}\right)\left(1-q_{i}^{U}\right)+\left[1-r_{i}\left(1-m_{i}\right)\right]\left(1-q_{i}^{A}\right)\right\}\\ & +p_{i}^{\mathrm{AS}}\left\{\delta \left(1-m_{i}\right)\left(1-q_{i}^{U}\right)+\left[1-\delta \left(1-m_{i}\right)\right]\left(1-q_{i}^{A}\right)\right\} \end{array} \end{array}$$

by adding Equations (8) and (10) to Equation (13). When $\beta^{U}$ is close to the threshold $\beta_{c}^{U}$, the probability of nodes being infected is close to zero; therefore, in this case, Equations (11) and (12) can be approximated as

(15) $$\begin{array}{c} q_{i}^{U}\approx 1-\beta^{U}\sum_{j}b_{ji}\epsilon_{j}=1-\sigma_{i}, \end{array}$$

and

(16) $$\begin{array}{c} q_{i}^{A}\approx 1-\gamma \beta^{U}\sum_{j}b_{ji}\epsilon_{j}=1-\gamma \sigma_{i}, \end{array}$$

where $p_{i}^{I}=\epsilon_{i}\ll 1$, and

(17) $$\begin{array}{c} \sigma_{i}=\beta^{U}\sum_{j}b_{ji}\epsilon_{j}. \end{array}$$

Then, substituting Equations (15) and (16) into Equation (14), we can obtain

(18) $$\begin{array}{c} \begin{array}{cc} \epsilon_{i}= & \epsilon_{i}\left(1-\mu \right)\\ & +p_{i}^{\mathrm{US}}\left\{r_{i}\left(1-m_{i}\right)\sigma_{i}+\left[1-r_{i}\left(1-m_{i}\right)\right]\gamma \sigma_{i}\right\}\\ & +p_{i}^{\mathrm{AS}}\left\{\delta \left(1-m_{i}\right)\sigma_{i}+\left[1-\delta \left(1-m_{i}\right)\right]\gamma \sigma_{i}\right\}. \end{array} \end{array}$$

In addition, as $\epsilon_{i}=p_{i}^{\mathrm{UI}}+p_{i}^{\mathrm{AI}}\ll 1$, then $p_{i}^{U}$, and $p_{i}^{A}$ can be approximated as

(19) $$\begin{array}{c} p_{i}^{U}=p_{i}^{\mathrm{US}}+p_{i}^{\mathrm{UI}}\approx p_{i}^{\mathrm{US}} \end{array}$$

and

(20) $$\begin{array}{c} p_{i}^{A}=p_{i}^{\mathrm{AS}}+p_{i}^{\mathrm{AI}}\approx p_{i}^{\mathrm{AS}}, \end{array}$$

respectively. Substituting Equations (19) and (20) into Equation (18) leads to

(21) $$\begin{array}{c} \begin{array}{cc} \epsilon_{i}= & \epsilon_{i}\left(1-\mu \right)\\ & +\left[p_{i}^{U}r_{i}\left(1-m_{i}\right)+p_{i}^{A}\delta \left(1-m_{i}\right)\right]\sigma_{i}\\ & +\left\{p_{i}^{U}\left[1-r_{i}\left(1-m_{i}\right)\right]+p_{i}^{A}\left[1-\delta (1-m_{i})\right]\gamma \sigma_{i}\right\}. \end{array} \end{array}$$

Then, removing the $O(\epsilon_{i})$ terms in Equations (7) and (9), and combining Equations (19) and (20), we obtain

(22) $$\begin{array}{c} p_{i}^{U}=p_{i}^{U}r_{i}\left(1-m_{i}\right)+p_{i}^{A}\delta \left(1-m_{i}\right) \end{array}$$

and

(23) $$\begin{array}{c} p_{i}^{A}=p_{i}^{U}\left[1-r_{i}\left(1-m_{i}\right)\right]+p_{i}^{A}\left[1-\delta \left(1-m_{i}\right)\right]. \end{array}$$

Thus, substituting Equations (22) and (23) into Equation (21), we can obtain

(24) $$\begin{array}{c} \begin{array}{cc} \epsilon_{i} & =\left(1-\mu \right)\epsilon_{i}+p_{i}^{U}\sigma_{i}+p_{i}^{A}\gamma \sigma_{i}\\ & =\left(1-\mu \right)\epsilon_{i}+\left(p_{i}^{U}\sigma_{i}+p_{i}^{A}\gamma \right)\beta^{U}\sum_{j}b_{ji}\epsilon_{j}. \end{array} \end{array}$$

Denoting $\delta_{ij}$ as the element of the identity matrix, Equation (24) can be rewritten as

(25) $$\begin{array}{c} \sum_{j}\left[\beta^{U}\left(p_{i}^{U}+\gamma p_{i}^{A}\right)b_{ji}-\mu \delta_{ij}\right]\epsilon_{j}=0. \end{array}$$

Defining matrix *H* with elements

(26) $$\begin{array}{c} h_{ij}=\left(p_{i}^{U}+\gamma p_{i}^{A}\right)b_{ji}, \end{array}$$

nontrivial solutions of Equation (25) are associated with eigenvectors of the matrix *H*, for which the most prominent real eigenvalues equal $\mu /\beta^{U}$. As a result, the epidemic threshold can be ascertained by the largest real eigenvalue of matrix *H*, i.e.,

(27) $$\begin{array}{c} \beta_{c}^{U}=\frac{\mu }{\Lambda_{max}\left(H\right)}. \end{array}$$

## 4. Simulation

In this section, we first validate the accuracy of the theoretical predictions, by comparing the results of the Microscopic Markov Chain Approximation (MMCA) approach to the Monte Carlo (MC) simulations. Then, we explore the impact of the three main parameters in the proposed model, including the infection attenuation factor γ (which influences the immunity level of the aware nodes), the mass media broadcast proportion η, and the inter-layer degree correlation $r_{s}$.

For the configuration of the multiplex network, we employed a scale-free network in the epidemic spreading layer, consisting of 1000 nodes and 2500 edges. The network exhibited a degree exponent of 2.5, and an average degree of 5. Considering that virtual social networks in reality are often denser than physical contact networks, we used a scale-free network with the same nodes and degree exponent in the information diffusion layer; however, the average degree of the information diffusion layer network was set to 10—higher than that of the disease spreading layer network.

Additionally, individuals’ behavior in the virtual network may differ from their behavior in real life, such as individuals who are active in the information layer network but have minimal contact with others in reality. To simulate such complex scenarios, we adjusted the inter-layer degree correlation $r_{s}$ of the dual-layer network. In our simulation experiments, we considered three cases: $r_{s}=1$; $r_{s}=-1$; and $r_{s}=0$. When $r_{s}=1$, the inter-layer degree correlation of the dual-layer network was fully positive, meaning that the high-degree nodes in the information diffusion layer were also high-degree nodes in the disease spreading layer. When $r_{s}=-1$, the high-degree nodes in the information diffusion layer corresponded to low-degree nodes in the disease spreading layer. When $r_{s}=0$, there was no correlation between the degrees of nodes in different layers of the dual-layer network. All the simulation results in this section were obtained by performing over 1000 independent simulations, and averaging the results. The initial proportion of infected nodes in the spreading process was set to 0.2, and the self-aware probability κ was set to 0 for all simulations.

**Figure 3:**
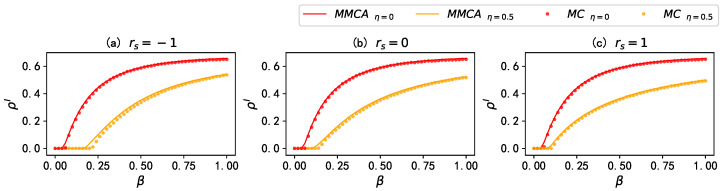
Comparison of the stationary fraction of infected individuals $\rho^{I}$ obtained by Monte Carlo (dotted line) simulations to the MMCA approach (solid line); $\rho^{I}$ versus β on multiplex networks with (**a**) $r_{s}=-1$, (**b**) $r_{s}=0$, and (**c**) $r_{s}=1$. The red lines and yellow lines denote, respectively, the results when η=0 and η=0.5. Values for the other parameters are set as: γ=0.4,λ=0.15, and μ=0.5.

Firstly, we examine the accuracy of the MMCA theory framework in predicting the stationary fraction of infected individuals, $\rho^{I}$. Figure 3 compares the fraction of $\rho^{I}$ obtained through the MMCA theory and MC simulations under different scenarios. It can be seen that, in the multiplex networks with various inter-layer degree correlations $r_{s}$, the MMCA theory accurately predicts the stationary fraction of infected individuals for the entire range of the considered infection rate β. Furthermore, by comparing the results with and without mass media propagation, the MMCA method accurately predicts the role of mass media in suppressing disease spreading.

**Figure 4:**
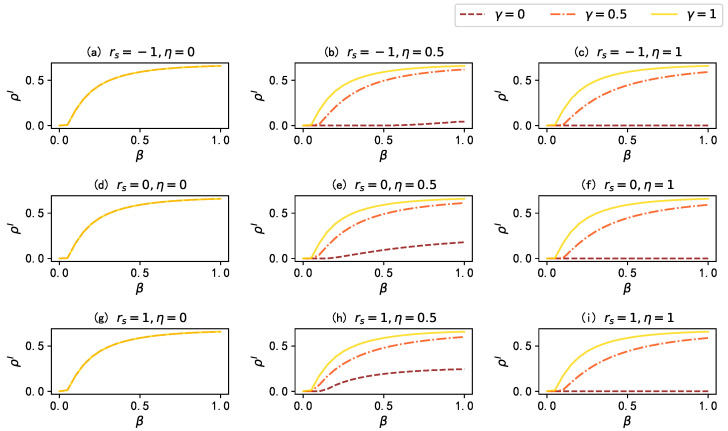
The stationary fraction of infected individuals $\rho^{I}$ versus the infection rate β for different values of the infection attenuation factor γ under different conditions. The brown dashed lines, orange dash–dot lines, and yellow solid lines denote the results when γ=0, γ=0.5, and γ=1, respectively.Values for the other parameters are set as λ=0.15, δ=0.6, and μ=0.5.

**Figure 5:**
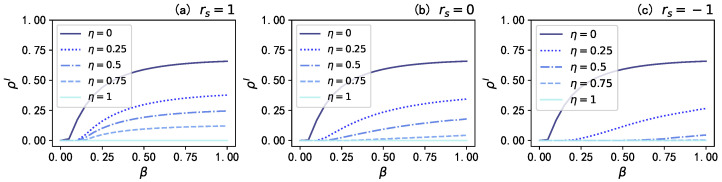
The stationary fraction of infected individuals $\rho^{I}$ as a function of the infection rate β for different mass media broadcast proportion η under different inter-layer degree correlations $r_{s}$. The lines colored from dark to light denote the results when η=0, η=0.25, η=0.5, η=0.75, and η=1, respectively. Values for the other parameters are set as: γ=0, μ=0.5, λ=0.15, and δ=0.6.

Secondly, we investigate the impact of two key dynamic parameters in the proposed model: namely, the infection attenuation factor γ and the mass media broadcast proportion η. Here, the infection attenuation factor γ regulates the probability of infection for aware state nodes ($\beta^{A}=\gamma \beta^{U}$), and the mass media broadcast proportion η determines the fraction of nodes influenced by mass media. Figure 4 illustrates the influence of the infection attenuation factor γ on the stationary fraction of infected individuals $\rho^{I}$ in different scenarios. The results in Figure 5 show that when there is no mass media broadcast (η=0), there is no information dissemination in the network: thus, the infection attenuation factor γ has little impact on $\rho^{I}$; however, when η=0.5, a smaller value of the infection attenuation factor γ leads to a stronger influence of information diffusion on disease spreading, resulting in a lower $\rho^{I}$. Moreover, when we propagate to all nodes in the information layer (η=1), the disease propagation is effectively suppressed; especially when the infection attenuation factor γ=0 (i.e., when aware state nodes are completely immune to the disease), the density of the stationary fraction of infected individual $\rho^{I}$ remains close to 0. These results indicate that in the proposed model, an increase in the infection attenuation factor γ significantly affects the final infection density of the disease, and a larger mass media broadcast proportion η enhances the inhibitory effect of increasing γ on the spread of the epidemic.

Furthermore, we also investigate the impact of varying the mass media broadcast proportion η on the stationary fraction of infected individuals $\rho^{I}$, when γ=0. As shown in Figure 5, in networks with different inter-layer degree correlations, increasing the value of η can effectively reduce $\rho^{I}$.

**Figure 6:**
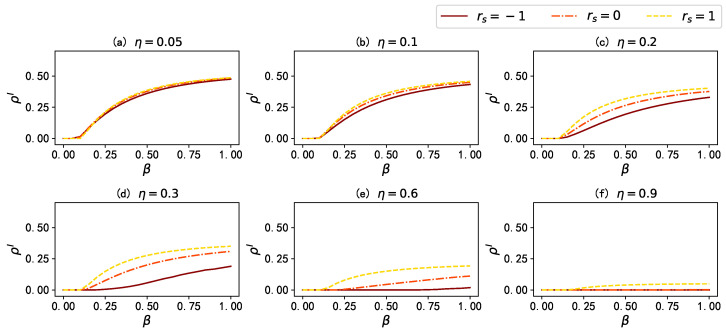
The stationary fraction of infected individuals $\rho^{I}$ as a function of the infection rate β for different inter-layer degree correlations $r_{s}$ under different mass media broadcast proportion η. The results are denoted by the brown solid lines, orange dash-dot lines, and yellow dashed lines when $r_{s}=-1$, $r_{s}=0$, and $r_{s}=1$, respectively. Other model parameters are set to be γ=0, μ=0.5, λ=0.15, and δ=0.6.

Finally, we discuss the impact of inter-layer degree correlation on the dynamics of the proposed model. Figure 6 illustrates the variation of the stationary fraction of infected individuals $\rho^{I}$ with the mass media broadcast proportion η in the multiplex networks with different inter-layer degree correlations $r_{s}$. Without loss of generality, we set the infection attenuation factor γ to 0. The results show that when the mass media broadcast proportion η is the same, in the case of $r_{s}=-1$ ($r_{s}=1$), the disease has the smallest (largest) $\rho^{I}$: this is because, when γ=0, once a node receives information from mass media, it cannot be infected anymore, which is equivalent to cutting the node off from other nodes in the disease spreading layer. In view of this, we supplemented a simulation experiment, by removing all connected edges of nodes with degree ranking within η in the disease spreading layer, and then observing the fragmentation of the network.

**Figure 7:**
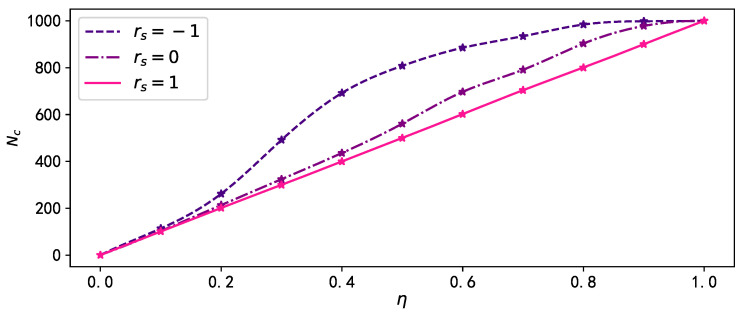
The number of connected components $N_{c}$ after removing all connected edges of nodes with degree ranking within mass media broadcast proportion η in the disease spreading layer. The pink solid line, purple dash-dot line, and purple dashed line denote the results when $r_{s}=1$, $r_{s}=0$, and $r_{s}=-1$, respectively.

As shown in Figure 7, as η increases, the number of connected components $N_{c}$ in the multiplex network with negative inter-layer degree correlation increases faster, leading to a higher likelihood of being fragmented into multiple “islands”, and limiting the spread of the disease in the network. The situation is reversed for networks with positive inter-layer degree correlation. These phenomena also explain the impact of inter-layer degree correlations on the disease spreading threshold.

**Figure 8:**
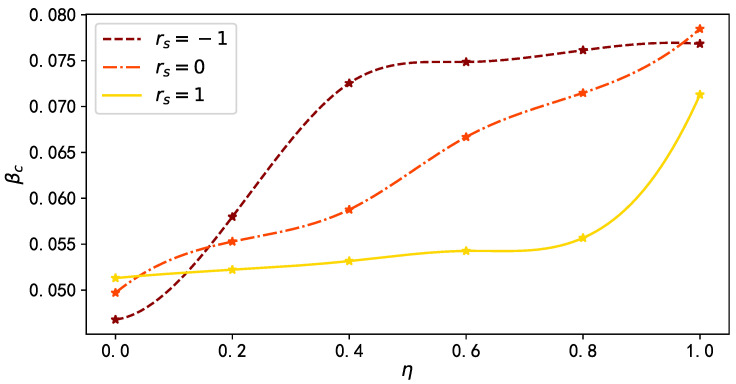
The epidemic threshold $\beta_{c}$ as a function of the mass media broadcast proportion η in multiplex network with different inter-layer degree correlations $r_{s}$. The brown dashed line, orange dash–dot line, and yellow solid line denote the results when $r_{s}=-1$, $r_{s}=0$, and $r_{s}=1$, respectively. Other model parameters are set as: γ=0, μ=0.5, λ=0.15, and δ=0.6.

As shown in Figure 8, when $r_{s}=-1$, as the proportion of mass media broadcast proportion η increases, the epidemic threshold $\beta_{c}$ rises rapidly, while for $r_{s}=1$, it takes a larger value of η for $\beta_{c}$ to show a significant increase.

## 5. Conclusions

During an epidemic, the diffusion of disease-related information can enhance individual awareness, and effectively curb the spread of the epidemic. Leveraging mass media to broadcast disease-related information is an effective approach to enhancing individual protection awareness. Considering the practical constraints of comprehensive promotion, which requires significant social resources, this study proposes a coupled information–epidemic spreading model with selective mass media. In this model, mass media can selectively target a certain proportion of high-degree nodes for information dissemination. We developed a Markov chain method for theoretical analysis of the proposed model, and examined the impact of three key model parameters—namely, the infection attenuation factor γ, the mass media broadcast proportion η, and the inter-layer degree correlation $r_{s}$ on the coupled dynamics.

Extensive simulations and detailed theoretical analyses consistently demonstrated that targeted promotion by mass media, to a certain proportion of high-degree nodes in the network, significantly reduces the infection density of the epidemic, and raises the epidemic threshold. Moreover, a higher promotion proportion leads to a stronger suppression effect on the epidemic. In addition, increasing the infection attenuation factor γ significantly affects the stationary fraction of infected individuals $\rho^{I}$, and a larger value of γ, combined with a higher proportion of mass media broadcast η, exhibits a more pronounced inhibitory effect on the epidemic spread. Finally, we analyzed the influence of the inter-layer degree correlation $r_{s}$ on the spreading dynamics. We found that, for a given proportion of mass media broadcast η, the inter-layer degree correlation $r_{s}=-1$ ($r_{s}=1$) results in the smallest (largest) outbreak size of the disease. Additionally, in the case of $r_{s}=-1$, the epidemic threshold $\beta_{c}$ increases rapidly with the increase of η, while for $r_{s}=1$, a visible increase in $\beta_{c}$ requires a larger value of η. These findings indicate that in a multiplex network with negative inter-layer degree correlation, the inhibitory effect of mass media promotion on epidemic spread is superior to cases with positive or no inter-layer degree correlation.

In summary, this study systematically investigated the coupled information–epidemic spreading model with selective mass media. The results provide a comprehensive understanding of the impact of selective broadcast mechanisms in coupled information–disease dynamics, and offer an important theoretical reference for epidemic prevention and control on the basis of disease-related information dissemination in reality. For future research, it would be worthwhile to extend our model to temporal multiplex networks, as both virtual-contact and physical-contact networks can change over time. Other, more realistic, network structures (for instance, hyper networks) and dynamic mechanisms (for example, social reinforcement effects) are also worth exploring, based on our model.

## Data Availability

Not applicable.
